# Cultural Adaptations of Healthcare Interventions: A Step‐by‐Step Guide

**DOI:** 10.1111/hex.70639

**Published:** 2026-03-19

**Authors:** Ed Breckin, Raabia Sattar, Nazreen Butt, Beth Fylan, Charles Vincent

**Affiliations:** ^1^ NIHR Yorkshire and Humber Patient Safety Research Collaboration Bradford United Kingdom; ^2^ NIHR Yorkshire and Humber Patient Safety Research Collaboration Lay leader Bradford United Kingdom; ^3^ University of Bradford. NIHR Yorkshire and Humber Patient Safety Research Collaboration Bradford United Kingdom; ^4^ University of Oxford Oxford United Kingdom

**Keywords:** cultural adaptation, ecological validity model, health equity, healthcare interventions, implementation science

## Abstract

**Introduction:**

Cultural adaptations of healthcare interventions are widely advocated to improve effectiveness, acceptability, and equity for diverse populations. Despite the existence of several cultural adaptation frameworks, there remains a lack of practical, transferable guidance on how adaptations are undertaken in applied healthcare settings. Adaptation processes are frequently underreported, limiting transparency, reproducibility, and learning across studies, especially within a UK context. This paper addresses this gap by presenting a structured, step‐by‐step guide for culturally adapting healthcare interventions, using a healthcare toolkit, ‘I Manage My Meds’, as a case study for adaptation.

**Methods:**

A methodological case study approach was used to develop an eight‐step, iterative guide for cultural adaptation. The guide integrates a phased structure model with the substantive domains of the Ecological Validity Model (EVM). Development was informed by collaboration with community stakeholders, patients, public contributors, translators, and researchers during the cultural adaptation of an existing healthcare intervention for older adults from a South Asian background in the UK. The guide was refined through repeated cycles of stakeholder engagement, pilot testing, and reflection, with adaptations systematically mapped to the eight EVM domains.

**Results:**

The resulting step‐by‐step guide provides practical direction on how to plan, implement, document, and refine cultural adaptations across a healthcare intervention. Key reflections from the guide are that cultural adaptations should be considered as cyclical processes rather than linear; deep‐level adaptations often require reframing intervention assumptions; and sustained stakeholder collaboration is essential for maintaining intervention fidelity while improving cultural relevance. The guide is designed to be transferable across populations, settings, and intervention types.

**Conclusion:**

This paper contributes practical methodological guidance to an underdeveloped area of implementation research. By offering a transparent and replicable step‐by‐step guide, it supports researchers and practitioners to move beyond superficial adaptations and to more consistently document cultural adaptation processes. Wider use of this guide may improve the quality, equity, and reproducibility of culturally adapted healthcare interventions.

**Patient or Public Contribution:**

The guide was developed in collaboration with the Leeds Older People's Forum and community representatives from a South Asian background who formed a stakeholder group. Alongside our lay leader expert, a co‐author on the paper, they contributed to reviewing intervention content, identifying culturally relevant adaptations, testing pilot materials, and refining the step‐by‐step guide through iterative feedback and collaboration.

## 1 Introduction

Cultural adaptation is defined as a methodological modification of an intervention that considers a user's needs, including their language, culture and values [1, 2]. Cultural adaptations generally take on two forms of adaptation: surface structure and deep structure [3]. The surface structure approach often comprises of smaller or superficial changes to the content and/or delivery of the intervention [4] and this may include translating materials into a preferred language, matching facilitators to participants by ethnicity, and altering imagery, scenarios, or programme examples so that they reflect the lived realities of the target population [5]. Deep structure modifications take a more considered approach to the intervention and are designed to also address ‘core cultural values’ in relation to psychological, environmental and social norms [6, 7, 8].

Interventions seeking to make deep structure adaptations include modifications for Black households that have incorporated content related to racial socialisation, religious values, and culturally specific parenting norms [9, 10]. Evidence suggests that surface adaptations without deeper structural change may have limited impact: a synthesis of culturally adapted youth mental health programmes found that surface‐level changes produced outcomes comparable to control groups, leading the authors to argue that deep adaptations may be necessary when interventions do not sufficiently align with cultural norms [11]. In some cases, changes to language or imagery may be sufficient; in others, failure to address deeper cultural meanings risks reinforcing assumptions embedded in the original intervention [2]. This distinction is recognised beyond healthcare. In education and community prevention programmes, deep cultural adaptation has involved embedding community worldviews, family structures, and belief systems into programme logic rather than simply substituting culturally equivalent terms or symbols [12].

There is growing evidence and recognition that interventions to improve health and wellbeing are more effective when they are adapted to the needs and culture of specific groups of people and to particular settings, such as primary or community care [13, 14, 15]. Interventions that are simply replicated are less likely to reproduce effective outcomes than those which are adapted to recognise a fit between intervention and context [16]. Adaptations also serve to reduce inequalities which may be generated by interventions, ensuring that they are delivered at a population level to the needs of previously excluded groups [16].

In practice, however, relatively few health interventions have been adapted to the needs of different groups. A recent narrative review of evidence from the National Health Service Diabetes Prevention Programme (NHS DPP) highlighted that despite existing recommendations to adapt interventions to local populations, the needs of diverse populations, particularly minority ethnic groups were not being met [17]. When cultural adaptations were made, they were often restricted to relatively superficial modifications, such as providing supportive materials in different languages, without considering wider problems of accessibility and access [18]. In addition, studies that evaluated cultural adaptations seldom reported the process of adaptation itself [19, 20].

As cultural adaptation research has increased over the past 20 years [21] there has also been a corresponding rise in published cultural adaptation frameworks. The majority of frameworks originated in the USA and mostly concern psychological or mental health interventions [20]. While a number of frameworks for cultural adaptation exist, few provide detailed practical guidance on how to conduct adaptation processes in applied contexts, particularly within the UK [21]. However, while frameworks are available, the actual process of culturally adapting an intervention is not typically described in detail within research [22]. In Yim and Schmidt's [23] recent review, only one study explicitly detailed the process of cultural adaptation using a structured framework [24].

In summary, there is growing evidence that cultural adaptation of health interventions is critical; there are a number of cultural adaptation frameworks available but almost no practical guidance on the actual process of cultural adaptation. In this paper, we share our own experience of adapting an effective intervention (the ‘I Manage My Meds Toolkit’) for a specific population and culture. We describe the intervention itself in its original context, the reasons for selecting particular frameworks and the steps we went through to engage the relevant communities and adapt the intervention at both surface and deeper levels. Finally, we offer some reflections and suggestions to support future cultural adaptation of future interventions.

### 1.1 The ‘I Manage My Meds’ Toolkit

**Figure 1:**
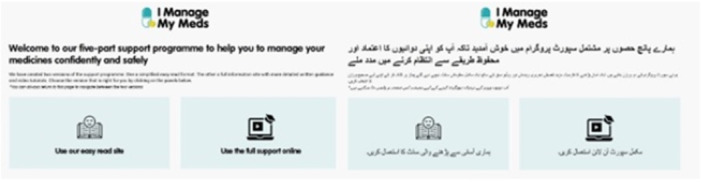
‘I Manage My Meds’ landing page and Urdu translation.

The *‘I manage my meds’* (IMMM) toolkit is a co‐designed intervention developed to support older adults living with frailty and managing polypharmacy at home [25]. Created using research‐embedded Experience‐Based Co‐Design (EBCD) [26] the toolkit draws directly on the lived experiences of older patients, family carers, and healthcare professionals to address the real‐world challenges of medicines self‐management. Through iterative development and stakeholder engagement, the intervention was shaped around three core priorities: day‐to‐day practical management, navigating healthcare systems, and improving communication with professionals (Figure 1).

Often with adaptations, the homogenising process means that healthcare interventions become more diffuse and less targeted [27]. As a result, the cultural adaptation of ‘I manage my meds’ was targeted at the South Asian population (people from India, Pakistan, Bangladesh, Sri Lanka and Nepal living in the UK). The South Asian population is the largest minority population in the United Kingdom [28] and Bradford, West Yorkshire, where our study took place, has an Asian or British Asian population of 32.1%, according to the most recent census [29]. Bradford has the second highest percentage of people nationally who identify as Pakistani (25.5%) and therefore translation efforts were focused on Urdu. People from a South Asian background face higher odds of multimorbidity [30] and multiple long‐term health conditions [31] both which require adequate medication management.

## 2 The Process of Cultural Adaptation

### 2.1 Designing the Process

While there is little detailed guidance on the cultural adaptation of interventions, the Cultural Adaptation (CAP) model provides a useful starting point and overall structure [32]. While other adaptation frameworks exist (e.g., Cultural Sensitivity Framework [2]. Hybrid Prevention Programme Model [33]), not all frameworks provide relevant process steps. We selected the CAP model because it provides a scaffold that foregrounds community collaboration and iterative piloting, which is absent from the two previously mentioned alternatives [34]. CAP is not a comprehensive adaptation framework, but it does provide a useful structural base onto which the Ecological Validity Model could be layered to provide cultural specificity. The CAP model proposes three phases: (1) researcher and community stakeholder collaboration, (2) piloting of the intervention, and (3) integrating observations into the adapted intervention. We followed this broad approach but in practice the three phases are a simplification of a much more complex and iterative process. In practice, as we describe below, our engagement and collaboration was a continuous collaborative process with repeated engagement, discussion, testing and refinement throughout the process. We suggest that while the CAP model is a useful foundation, that it is best seen as a series of cycles of change and refinement rather than a simply a three‐phase model.

### 2.2 Selecting an Adaptation Framework

We selected the Ecological Validity Model (EVM) [35] to guide our cultural adaptation. A recent review by Weeks [36] exploring the effectiveness of theory‐based strategies in cultural adaptation processes found the EVM to be among the most effective frameworks to utilise. The EVM has been highly cited [37] and used in a wide variety of contexts including Chinese dementia patients [38] mental health and substance abuse interventions [39] e‐learning programmes [40] and Latinx communities [41].

The EVM provides a systematic method for documenting cultural adaptations through its eight comprehensive domains: language, people, metaphor, content, concepts, goals, methods, and contexts [42]. This framework seeks to understand individuals and groups within their cultural, social, and political spheres, aiming to ‘culturally centre’ an intervention by ensuring all eight domains are incorporated for the intervention to go beyond surface level adaptation.

The EVM offers a comprehensive process for understanding the community and identifying which specific components of an intervention require adaptation. This differs from many cultural adaptation frameworks that fail to clearly indicate which intervention components should be adapted [20]. The EVM, unlike many adaptation frameworks, provides guidance on the relevant domains to consider in the adaptation process, which allows the collaborative research team to examine the components of the intervention across all eight domains to assess where adjustments are needed. All eight dimensions of the EVM were discussed at each stakeholder meeting, guided our review of the intervention components, and it also determined what aspects of IMMM required modification to ensure cultural appropriateness and relevance.

## 3 A Step‐by‐Step Guide to Culturally Adapting Healthcare Interventions

Drawing from the existing cultural adaptation frameworks described above and our experience of undertaking an adaptation, this section presents an eight‐step, iterative guide for culturally adapting healthcare interventions. The steps in the adaptation process emerged through iterative reflection on the adaptation process as it happened in the IMMM adaptation. After cycles of stakeholder engagement, piloting, and refinement, the activities were mapped and consolidated into eight steps, which together encompassed all central activities in the IMM adaptation. These were then framed in more general terms, further refined and checked against the Ecological Validity model, to form the eight general steps described below. The guide is intended to be transferable across interventions, settings, and populations. While the steps are presented sequentially for clarity, cultural adaptation is rarely linear; steps may overlap, repeat, or occur concurrently depending on the context, resources, and stakeholder input and expectations. The process integrates the phased logic of the Cultural Adaptation Process (CAP) model with the substantive domains of the Ecological Validity Model (EVM), thereby offering both structural guidance and practical direction on what to adapt and how.

An important consideration before Step 1, akin to ‘Step 0’, is that adaptation should not occur post hoc but be embedded in intervention development. The National Health Service (NHS) ‘Our Strategy’ focuses on ensuring that health and social care research is done with and for everyone [43]. Similarly, the National Institute for Health Research (NIHR) suggest that that all those impacted by the outcomes of research investigations should be also involved [44]. Research should ensure that individuals from all backgrounds are encouraged to be involved in research study conception, design, and through to dissemination.

**Step 1: Establish a collaborative adaptation group**

The first step is to establish a collaborative group that brings together researchers, members of the target population, community or third‐sector representatives, and/or relevant policy or practice stakeholders. The purpose of this group is to ensure that the adaptation process is co‐produced, culturally grounded, and informed by lived experience right from the initial stages of planning.

Key considerations at this stage include ensuring appropriate representation across age, gender, language, and lived experience, and identifying bilingual or bicultural members where possible. Clarity around the aims and rationale for adaptation should happen at this stage to encourage engagement and buy‐in from your collaborative group. This step also involves establishing boundaries around intervention fidelity to ensure that core mechanisms of action are preserved during adaptation.

**Step 2: Select and operationalise an adaptation framework**

A cultural adaptation framework should be selected to guide the process in a systematic and transparent way. Frameworks such as the Ecological Validity Model (EVM) are particularly useful as they specify domains that require consideration, including language, people, metaphors, content, concepts, goals, methods, and context. The framework, such as EVM, can guide discussion points throughout co‐design stages. At this stage, the framework domains can be translated into review questions that can be used consistently across meetings and stages. The adaptation framework should be revisited iteratively, rather than adoption as a one‐off checklist, supporting ongoing reflection on cultural relevance throughout the process.

**Step 3: Initial review of the intervention**

The original intervention and/or toolkit should then be reviewed collaboratively with the adaptation group. Using the selected framework, stakeholders can systematically examine all domains of the intervention to identify where and if cultural mismatches exist.

It is important at this initial stage that the group distinguishes between surface structure adaptations (e.g. translation, imagery, formatting) and deep structure adaptations (e.g. values, family roles, social norms, or assumptions embedded within the intervention). An output at this stage could be producing a list of proposed adaptations mapped to the domains of your chosen framework, alongside documentation of the rationale for each proposed change.

**Step 4: Create a pilot version of the adapted intervention**

Findings from step 3's review can then be translated into an initial iteration of the adapted intervention. This may involve producing translated materials, modifying imagery or examples, changing formats or delivery modes, or adding content that better reflects cultural practices and values.

Throughout this step, maintaining fidelity to the core components and mechanisms of the original intervention is essential. All adaptations should be documented, including what was changed, the rationale for the change, and which framework domain informed the decision.

**Step 5: Check and refine the pilot version using the adaptation framework**

The pilot version should now be reviewed with the collaborative group to assess whether the adaptations implemented adequately address both surface and deep cultural elements. The adaptation framework should be revisited, with each domain discussed to identify inconsistencies or unintended consequences due to adaptation. This step may result in further refinements to language, content, imagery, or framing, ensuring that the intervention remains coherent.

**Step 6: Test the pilot version with the target population**

The refined pilot intervention is then tested with members of the target population. This testing may take the form of acceptability studies, usability testing, feasibility work, or qualitative feedback sessions, depending on resources and study aims.

Data collection methods should themselves be culturally appropriate, and feedback should be sought not only from participants but also from those involved in recruitment or delivery, such as community researchers or facilitators.

**Step 7: Further refinement and iteration**

Findings from pilot testing are used to further refine the intervention. This step emphasises iteration. Changes are made, reviewed against the chosen adaptation framework, and where necessary (and if feasible) discussed again with collaborators.

Step 7 may be repeated across multiple cycles if required. This may be necessary when working with complex interventions or diverse populations. Transparent documentation of these iterations is essential to support reproducibility and reporting.

**Step 8: Formal testing in a wider population**

The final step involves formal evaluation of the adapted intervention in a wider population. This may include but is not limited to feasibility, process, or effectiveness studies. At this stage, researchers should consider whether study procedures, outcome measures, and implementation strategies require further cultural adaptation. Findings from formal testing may prompt additional refinements, reinforcing the cyclical nature of cultural adaptation.

### 3.1 Cultural Adaptation of the ‘I Manage My Meds’ Toolkit

To illustrate how this process operates in practice, the following section presents a worked example describing the cultural adaptation of the ‘I Manage My Meds’ toolkit for older adults from a South Asian background living in the UK. Each stage of the adaptation is mapped onto the eight steps outlined above (Table 1). The specific adaptations undertaken across each of the eight Ecological Validity Model domains are summarised in Table 2. To further clarify the distinction between surface and deep structural adaptation, illustrative examples from the IMMM adaptation process are presented in Box 1.

**Table 1:** Eight step adaptation process for ‘I Manage My Meds’: steps and research activities.

Stage of adaptation	Example activities from the ‘I Manage My Meds’ adaptation
Step 1 – Establish collaborative adaptation group	Established a collaborative agreement with the Leeds Older People's Forum to assess the toolkit and plan an easy‐read format. The easy‐read version was developed to improve accessibility for a wider audience and ease non‐digital translation Convened a stakeholder group including older adults from a South Asian background and representatives from local community networks. Meetings explored culturally specific practices related to medication management, including family involvement, communication norms with healthcare professionals, religious considerations, and preferred formats for health information. Insights from these discussions informed both surface and deep structural adaptations of the toolkit.
Step 2 – Select and operationalise adaptation framework	Selected the Ecological Validity Model. Agreement to use all eight domains iteratively to guide discussions and framework was operationalised as review questions for future meetings
Step 3 ‐ Initial review of the intervention	Met with the Leeds Older People Forum and our newly established South Asian stakeholder group to review the toolkit content to identify potential surface and deep cultural adaptations Designed an easy‐read format with an organisation experienced in improving intervention accessibility and inclusivity.
Step 4 – Create pilot adapted version	Developed an easy‐read version of the toolkit Collaborated with Bradford Talking Media to develop all materials to incorporate feedback and improve accessibility of the toolkit Hired an Urdu translation company to dub all videos of the toolkit into Urdu so videos can be interchangeably played in Urdu or English Translated all intervention materials into Urdu Imagery and content amended following feedback from both collaborative groups.
Step 5 – Check and refine pilot using framework	Reviewed the translated research material with the certified Urdu speaker and translator Revisited the Leeds Older People Forum group to review the easy‐read format developed from their input Reviewed the easy‐read version of the toolkit with the stakeholder advisory group from a South Asian background to check the cultural competency of the materials Reviewed the adapted materials with stakeholder groups using EVM domains; refinement of language, imagery, and content
Step 6 – Test pilot version	Acceptability study of ‘I manage my meds’ targeting both a general population and individuals from a South Asian background. English and Urdu versions of the toolkit offered to participants based on their preferences Sought feedback on linguistic adaptations and cultural relevance of images and videos in the toolkit via the acceptability questionnaire.
Step 7 – Further refinement and iteration	Iterative changes based on stakeholder feedback, acceptability study findings, translator checks, and community researcher input.
Step 8 – Formal testing in wider population	Acceptability findings taken back to Leeds Older People Forum and South Asian stakeholder group for further cyclical iterations and feedback. Potential wider testing and adaptation to other populations planned.

**Table 2:** Ecological validity model domains for ‘I Manage My Meds’.

Domains of ecological validity model (EVM)	Cultural adaptation
Language	Translating materials and videos to Urdu. Provision of subtitles in Urdu in both voice over versions of videos as stakeholder groups suggested that some individuals who utilise Urdu are proficient readers but less so with listening skills.
People	Ensuring that individuals from a South Asian background are included in images and videos in the toolkit. The original version of the toolkit was predominantly imagery featuring white British older adults alone managing medicines. The adapted imagery featured images of older adults from a diverse range of ethnicities. We also featured images to reflect multigenerational households and carers assisting with medication management. We accentuated the imagery to represent that medicine management is not a solely individualised activity.
Metaphors	Checking terminology to ensure that it all language used is culturally relevant and checking for idiom use. Due to the fact that we had developed an ‘easy‐read’ version of the toolkit, we sought to make the language accessible throughout but attention to metaphors should be checked to ensure clarity.
Content	Check all imagery, videos, and text are appropriate. Reframing the toolkit to speak to caregivers ‘looking after a family member's medications can be difficult’ rather than previous iteration ‘managing your own medications can be difficult’.
Concepts	Adding in additional text to reflect that medicine management may be undertaken by relatives and/or family members to reflect care at home for South Asian individuals. Original toolkit wording: ‘you manage your medicines independently.’ Adapted toolkit wording: ‘You and your family may share responsibility in managing your medicines.’ Printouts from the toolkit which help list active medications were adapted to be a family/carer shared goal to help with medicines management. This was to encourage family members/carers to attend GP and/or pharmacy appointments to facilitate that management is a shared responsibility.
Goals	Set the goal of the cultural adaptation to ‘turn the original ‘I manage my meds’ into a toolkit which matched the language and beliefs of the South Asian older adults managing their medicines at home.’ Medication management was framed in a culturally relevant manner. Encouraging the participant of family members to participate in the management to ensure the better outcomes for their family members managing medicines. Offering carers the opportunity to raise questions with the family member's GP and/or pharmacist via printouts like ‘questions to ask about my medications’. Adapting the toolkit into something that the family can utilise together to improve outcomes for older people from a South Asian background.
Methods	Development an easy‐read version of the toolkit to make it more accessible and also a hard copy so the option to utilise as web‐based or hard copy. Previous version was digital‐only. Hard copy version was accessible in English and/or Urdu.
Context	Accounting for how medicine management is undertaken by older people from a South Asian background. Approach to how medicines were managed became a shared value. This resulted in changes to the toolkit which referenced how medications should be stored, ordered, checked, and taken, and moved it away from ‘what you can do’ to a context that supported shared caregiving ‘what you and your family can do.

Box 1 Illustrative Examples of Surface and Deep Cultural Adaptations.

Example 1: Reframing Individual Self‐management of medicines as shared family responsibility (Deep Structural Adaptation)

Original intervention assumption: The original *I Manage My Meds* toolkit positioned medicine management primarily as an individual responsibility, reflecting a self‐management approach favourable with the original co‐design group that was largely White British.

Stakeholder insight: Stakeholder discussions with older adults from a South Asian background highlighted that medicine management was often undertaken collectively, with adult children or extended family members supporting organisation, reminders, and communication with healthcare professionals. Participants described caregiving as an expression of intergenerational responsibility rather than loss of independence.

Adaptation made: Sections of the toolkit were revised to explicitly acknowledge shared caregiving roles. Language was reframed from ‘managing your medicines independently’ to wording recognising collaborative management within families. Scenarios and examples were amended to depict multigenerational involvement, and prompts were added to encourage discussion between older adults and family members.

Why this represents deeper adaptation: This change altered the underlying conceptual framing of responsibility embedded in the intervention. Rather than reinforcing an individualised model of autonomy, the toolkit was adapted to align with collectivist caregiving norms and culturally embedded expectations of family support.

Example 2: Translation, dubbing, and visual representation (Surface Structural Adaptation)

Original intervention format: The toolkit was delivered in English and included videos and imagery predominantly reflecting White British older adults.

Adaptation made: All written materials were translated into Urdu, and videos were professionally dubbed to allow users to toggle between English and Urdu. Imagery was revised to include older adults and families from South Asian backgrounds. An easy‐read and printable format was also developed to increase accessibility.

Why this represents surface adaptation: These changes improved accessibility enabling participants to engage more thoroughly with the intervention materials. However, they did not alter the core conceptual assumptions of the IMMM.

Example 3: Conceptual similarity in language and communication (Deep Linguistic Adaptation)

Original wording: The original toolkit assumed high health literacy and direct, assertive communication with healthcare professionals in how to self‐manage medications.

Stakeholder insight: Participants highlighted variations in preferred communication styles, including highlighting how family members could defer to medical authority and hesitancy in questioning clinicians. Literal translation risked preserving assumptions about assertive self‐advocacy that may not align with the South Asian cultural norms.

Adaptation made: Language was revised to provide culturally sensitive prompts supporting discussion with healthcare professionals while respecting communication preferences. Translation was conducted collaboratively to ensure conceptual rather than literal translations, with attention to tone and clarity.

Why this illustrates deeper adaptation: Although involving linguistic change, this adaptation addressed underlying norms about authority, communication, and patient preferences when interacting with healthcare professionals, moving beyond translation alone.

## 4 Discussion

Cultural adaptations of healthcare interventions are widely recognised as essential for improving effectiveness and equity, particularly for minority and underserved populations [16, 45]. However, the detailed methods for *how* cultural adaptations are developed and implemented remain underreported, often treated as a ‘black box’ in the literature [46, 47].

Our process of adapting the ‘I manage my meds’ toolkit for older adults from a South Asian background demonstrates a systematic and replicable pathway for cultural adaptation. By involving community stakeholders of the target population, in our case older adults from a South Asian background, at every stage, the adaptation process attempted to improve relevance, ownership, and acceptability. This participatory approach aligns with recommendations for culturally appropriate healthcare design [32, 35] and demonstrates how co‐production can lead to interventions that reflect users’ lived experiences, values, and beliefs.

Several of the adaptations in our study extended beyond surface‐level translation. The study highlighted how medication management among older South Asian adults is often a collective, family‐based activity – contrasting with the individualised framing common in mainstream interventions [48]. We therefore modified the toolkit's structure to acknowledge shared caregiving roles and added language that validated family involvement. This dynamic represents a critical deep structure adaptation that aligns with South Asian collectivist care norms. The study considered the ethical obligation in respecting diverse cultural values and identities in healthcare interventions as the literature often overlooks intersectionalites and how this influences health behaviours and intervention effectiveness [48]. In terms of surface adaptations, the translation of materials and videos into Urdu was well received, particularly the dubbed videos, which addressed limitations in Urdu literacy among the target population. This supports findings from prior studies, which emphasise the importance of audio‐visual materials in overcoming language barriers in older populations [49].

We recommend involving stakeholders from the target population as early as possible; in this instance, it allowed us to understand the nuances in medication management in the South Asian community. The core team working on adaptation will include, but not limited to, members of the public or patient population, policy and practice stakeholders, and researchers. The experts on specific components in the adaptation process should be consulted on an ad hoc basis at relevant stages [16] to ensure adaptation and modification reflects the appropriate cultural nuances. This enables an environment that encourages different perspectives but retaining fidelity of the intervention echoing previous literature on cultural adaptation [50].

The research team also need to acquire ‘cultural competence’ in the sense of learning about and reflecting on the cultural knowledge, differences across cultures and strategies for meeting culturally specific needs [51]. Employing bilingual or bicultural staff enables this process [52]. Striving for cultural competence should be coupled with practicing cultural humility, recognising that cultural knowledge is never complete and requires ongoing reflection and power sharing with communities. Our community researchers were a great asset, providing understanding and cultural competency training for the whole research team [53, 54]. Our research team included members from both inside and outside the South Asian communities involved. Whilst some of the research team shared linguistic and/or cultural proximity, others approached the work as outsiders. We addressed this, and would recommend to others to do the same, by prioritising stakeholder leadership in decision‐making and treating the community expertise as primary rather than advisory.

Commensurate compensation and recognition of the input of all external experts is necessary in this process. To draw on linguistic preferences, cultural idioms, literacy considerations, and content, requires considerate input from individuals with lived experience and knowledge of the target population and budget should reflect the need to revisit and review with their expertise. Budgets need to also include translations of materials, costs of dubbing videos in new languages, changes to websites changes and further checks including time for those involved in reviewing the toolkit and its various iterations.

Suggestions for further research should begin with building on empirical evidence of these kinds of adaptations. It would also be pertinent to culturally adapt additional interventions in the UK following the same EVM‐guided process detailed in this paper to assess its effectiveness across a range of interventions. Similar adaptations utilising the EVM domains have been undertaken internationally [50, 55] but not within a UK setting. Future research should also address the extent to which social experiences, such as racism and discrimination, might limit uptake of culturally adapted interventions [18]. While cultural adaptations may enable a more culture‐sensitive strategy for intervention delivery, more could be done to explore how this could be delivered within a context that largely implements group‐based interventions.

Adaptation methods have not been previously reported and the lack of documentation of these processes add to blurring sources of bias when assessing and analysing factors of intervention efficacy [56]. As a result there is a lack of empirical evidence on the kinds of adaptations that lead to higher acceptability or efficacy of treatments [47]. Further adaptations, following our guide or differing models in the UK, can enable us to develop transparent criteria on how to culturally adapt (and document) healthcare interventions.

## 5 Conclusion

Cultural adaptations of healthcare interventions are crucial for enhancing their effectiveness and combating health disparities. This paper presented a transparent documentation and step‐by‐step account of the cultural adaptation of the ‘I manage my meds’ toolkit. Guided by the Ecological Validity Model (EVM), it sought to address a gap in the literature by providing a replicable pathway for future cultural adaptations. The paper demonstrated the importance of early and continuous involvement of advisory groups from a target population to improve the acceptability and relevance of interventions. With the need for further effectiveness studies, this work provides valuable guidance for future cultural adaptations in the UK context and contributes to the broader goal of achieving health equity through culturally sensitive healthcare design.

## Author Contributions

**Ed Breckin:** conceptualisation, writing – original draft, writing – review and editing, investigation, methodology. **Raabia Sattar:** writing – reviewing and editing, supervision. **Nazreen Butt:** conceptualisation, writing – reviewing and editing. **Beth Fylan:** funding acquisition, writing – reviewing and editing, supervision. **Charles Vincent:** conceptualisation, funding acquisition, writing – original draft, supervision.

## Ethics Statement

The guide was developed during a research study exploring acceptability of a healthcare intervention. That study received ethical approval: The University of Bradford Research Ethics Committee on 19/02/2024 and the approval number is E1165 The Health Research Authority on 21/03/2024 and the approval number is 24/HRA/0645.

## Conflicts of Interest

The authors declare no conflicts of interest.

## Data Availability

Data sharing not applicable to this article as no datasets were generated or analysed during the current study.

## References

[1] G. Bernal , M. I. Jiménez‐Chafey , and M. M. Domenech Rodríguez , “Cultural Adaptation of Treatments: A Resource for Considering Culture in Evidence‐Based Practice,” Professional Psychology: Research and Practice 40, no. 4 (2009): 361–368.

[2] K. Resnicow , T. Baranowski , J. S. Ahluwalia , and R. L. Braithwaite , “Cultural Sensitivity in Public Health: Defined and Demystified,” Ethnicity & Disease 9, no. 1 (1999): 10–21. 10355471

[3] E. V. Cardemil , “Cultural Adaptations to Empirically Supported Treatments: A Research Agenda,” Scientific Review of Mental Health Practice 7, no. 2 (2010): 8–21.

[4] V. L. Thompson , M. Johnson‐Jennings , A. A. Baumann , and E. Proctor , “Use of Culturally Focused Theoretical Frameworks for Adapting Diabetes Prevention Programs: A Qualitative Review,” Preventing Chronic Disease 12:E60 (2015): 7. 10.5888/pcd12.140421 PMC4436044 25950567

[5] J. Chu and A. Leino , “Advancement in the Maturing Science of Cultural Adaptations of Evidence‐Based Interventions,” Journal of Consulting and Clinical Psychology 85, no. 1 (2017): 45–57, 10.1037/ccp0000145. 28045287

[6] M. Barrera , F. G. Castro , L. A. Strycker , and D. J. Toobert , “Cultural Adaptations of Behavioral Health Interventions: A Progress Report,” Journal of Consulting and Clinical Psychology 81, no. 2 (2013): 196–205. 22289132 10.1037/a0027085 PMC3965302

[7] N. Mier , M. G. Ory , and A. A. Medina , “Anatomy of Culturally Sensitive Interventions Promoting Nutrition and Exercise in Hispanics: A Critical Examination of Existing Literature,” Health Promotion Practice 11, no. 4 (2010): 541–554. 19193933 10.1177/1524839908328991 PMC3780354

[8] L. Terragni , E. Beune , K. Stronks , et al., “Developing Culturally Adapted Lifestyle Interventions for South Asian Migrant Populations: A Qualitative Study of the Key Success Factors and Main Challenges,” Public Health 161 (2018): 50–58. 29902781 10.1016/j.puhe.2018.04.008

[9] K. van Mourik , M. R. Crone , M. S. de Wolff , and R. Reis , “Parent Training Programs for Ethnic Minorities: A Meta‐Analysis of Adaptations and Effect,” Prevention Science 18, no. 1 (2016): 95–105, 10.1007/s11121-016-0733-5. PMC5236066 27882498

[10] A. T. Clarke , G. E. Soto , J. Cook , C. Iloanusi , A. Akwarandu , and V. Still Parris., “Adaptation of the Coping With Stress Course for Black Adolescents in Low‐Income Communities: Examples of Surface Structure and Deep Structure Cultural Adaptations,” Cognitive and Behavioral Practice 29, no. Issue 4 (2022): 738–749, 10.1016/j.cbpra.2021.04.005. 36387782 PMC9642973

[11] N. A. Gonzales , A. S. Lau , V. M. Murry , A. A. Pina , and M. Barrera, Jr. “Culturally Adapted Preventive Interventions for Children and Adolescents,” In *Developmental psychopathology: Risk, resilience, and intervention*, ed. D. Cicchetti (John Wiley & Sons, Inc, 2016), 874–933. 10.1002/9781119125556.devpsy417.

[12] S. K. Okamoto , S. Kulis , F. F. Marsiglia , L. K. Holleran Steiker , and P. Dustman , “A Continuum of Approaches Toward Developing Culturally Focused Prevention Interventions: From Adaptation to Grounding,” Journal of Primary Prevention 35, no. 2 (2014): 103–112. 24322970 10.1007/s10935-013-0334-z PMC3943988

[13] O. Razum and J. Spallek , “Addressing Health‐Related Interventions to Immigrants: Migrant‐Specific or Diversity‐Sensitive?,” International Journal of Public Health 59, no. 6 (2014): 893–895. 25012801 10.1007/s00038-014-0584-4

[14] G. Divan , S. U. Hamdani , V. Vajartkar , et al., “Adapting An Evidence‐Based Intervention for Autism Spectrum Disorder for Scaling Up in Resource‐Constrained Settings: The Development of the Pass Intervention in South Asia,” Global Health Action 8, no. 1 (2015): 27278. 26243710 10.3402/gha.v8.27278 PMC4524889

[15] A. Dhir , P. Kaur , S. Hassan , and D. Vrontis , “Reimagining Our Futures Together: An Early Bird's‐Eye View of Inclusive Organizational Behavior,” Journal of Organizational Behavior 45, no. 9 (2024): 1397–1412.

[16] G. Moore , M. Campbell , L. Copeland , et al., “Adapting Interventions to New Contexts—The Adapt Guidance,” BMJ 374 (2021): n1679. 34344699 10.1136/bmj.n1679 PMC8329746

[17] C. Koning , M. Pelletier , and J. Spooner , “The National Health Service England Diabetes Prevention Program—A Narrative Review,” Journal of Diabetology 14, no. 4 (October 2023): 198–206.

[18] T. Katangwe‐Chigamba , K. Kantilal , J. Hartley‐Palmer , et al., “Diet and Physical Activity Interventions for People From Minority Ethnic Backgrounds in the UK: A Scoping Review Exploring Barriers, Enablers and Cultural Adaptations,” Journal of Racial and Ethnic Health Disparities 12, no. 5 (October 2025): 3024–3068. 39145834 10.1007/s40615-024-02112-y PMC12446416

[19] R. Baños , R. Herrero , and D. Vara , “What Is the Current and Future Status of Digital Mental Health Interventions?,” Spanish Journal of Psychology 25 (2022): e5. 35105398 10.1017/SJP.2022.2

[20] S. Day , K. Laver , Y. H. Jeon , K. Radford , and L. F. Low , “Frameworks for Cultural Adaptation of Psychosocial Interventions: A Systematic Review With Narrative Synthesis,” Dementia 22, no. 8 (2023): 1921–1949. 37515347 10.1177/14713012231192360 PMC10644683

[21] S. Rathod , L. Gega , A. Degnan , et al., “The Current Status of Culturally Adapted Mental Health Interventions: A Practice‐Focused Review of Meta‐Analyses,” Neuropsychiatric Disease and Treatment 14 Jan 4 (2018): 165–178. 29379289 10.2147/NDT.S138430 PMC5757988

[22] M. A. Kirk , J. E. Moore , S. Wiltsey Stirman , and S. A. Birken , “Towards a Comprehensive Model for Understanding Adaptations’ Impact: the Model for Adaptation Design and Impact (Madi),” Implementation Science 15, no. 1 (2020): 56. 32690104 10.1186/s13012-020-01021-y PMC7370455

[23] S. H. Yim and U. Schmidt , “The Effectiveness and Cultural Adaptations of Psychological Interventions for Eating Disorders in East Asia: A Systematic Scoping Review,” International Journal of Eating Disorders 56, no. 12 (2023): 2165–2188. 37726977 10.1002/eat.24061

[24] S. Hamatani , K. Matsumoto , T. Ishibashi , et al., “Development of a Culturally Adaptable Internet‐Based Cognitive Behavioral Therapy for Japanese Women With Bulimia Nervosa,” Frontiers in Psychiatry 13 (2022): 942936. 36081468 10.3389/fpsyt.2022.942936 PMC9446753

[25] G. Previdoli , R. Simms‐Ellis , J. Silcock , et al., “Supporting Older People Living With Frailty to Self‐Manage Multiple Medicines: An Experience‐Based Co‐Design of a Complex Intervention Developed in UK Primary Care,” Health Expectations 28, no. 5 (2025): e70364. 40916547 10.1111/hex.70364 PMC12415351

[26] B. Fylan , J. Tomlinson , D. K. Raynor , and J. Silcock , “Using Experience‐Based Co‐Design With Patients, Carers and Healthcare Professionals to Develop Theory‐Based Interventions for Safer Medicines Use,” Research in Social and Administrative Pharmacy 17, no. 12 (2021): 2127–2135. 34187746 10.1016/j.sapharm.2021.06.004

[27] L. Hiam , C. Nagpaul , L. Hazard , et al., No Future Without Equity: Imperatives for a Fair and Inclusive NHS (NHS Race and Health Observatory, 2024).

[28] Centre on the Dynamics of Ethnicity, Response to the Government's Commission on Race and Ethnic Disparities Report 2021, 2021, https://www.ethnicity.ac.uk/discover/briefings/sewell-report-response/ consulted 23 October 2023.

[29] City of Bradford Metropolitan District Council. Population [Internet] . Understanding Bradford District; [cited 2026 Jan 15]. Available from: https://ubd.bradford.gov.uk/about-us/population/.

[30] M. Ashworth , S. Durbaba , D. Whitney , J. Crompton , M. Wright , and H. Dodhia , “Journey to Multimorbidity: Longitudinal Analysis Exploring Cardiovascular Risk Factors and Sociodemographic Determinants in an Urban Setting,” BMJ Open 9, no. 12 (2019): e031649. 10.1136/bmjopen-2019-031649 PMC7008443 31874873

[31] B. Hayanga , M. Joshi , K. Hartley , et al. Healthcare Interventions to Improve Health Outcomes for Racially Minoritised People With Multiple Long‐Term Conditions: A Systematic Review and Narrative synthesis. 10.1186/s12889-026-28056-y PMC13445657 42321690

[32] M. M. Domenech Rodríguez , A. A. Baumann , and A. L. Schwartz , “Cultural Adaptation of an Evidence Based Intervention: From Theory to Practice in a Latino/A Community Context,” American Journal of Community Psychology 47, no. 1 (2011): 170–186. 21116707 10.1007/s10464-010-9371-4

[33] F. G. Castro , M. Barrera , and C. R. Martinez , “The Cultural Adaptation of Prevention Interventions: Resolving Tensions Between Fidelity and Fit,” Prevention Science 5, no. 1 (2004): 41–45. 15058911 10.1023/b:prev.0000013980.12412.cd

[34] J. Leung , S. Sekar , L. Madrigal , and C. Escoffery , “A Scoping Study of Cultural Adaptation Frameworks,” Health Promotion Practice 26, no. 4 (2025): 807–825. 39529278 10.1177/15248399241292317

[35] G. Bernal , J. Bonilla , and C. Bellido , “Ecological Validity and Cultural Sensitivity for Outcome Research: Issues for the Cultural Adaptation and Development of Psychosocial Treatments With Hispanics,” Journal of Abnormal Child Psychology 23, no. 1 (1995): 67–82. 7759675 10.1007/BF01447045

[36] A. Weeks , “Culturally Adapting Evidence‐Based and Informed Practices to Meet Client Population Needs and Ensure Appropriate Allocation of Scarce Resources: A Systematic Review,” Human Service Organizations: Management, Leadership & Governance 46, no. 3 (2022): 238–263.

[37] K. Z. Shum , E. Barry , S. M. Kiefer , et al., “Adapting a Positive Psychology Intervention Using the Ecological Validity Model: Process and Lessons Learned,” Contemporary School Psychology 29, no. 1 (2025): 168–187.

[38] K. L. Y. Wong and L. Hung , “Cultural Adaptation in Television Technology for Older Adults With Dementia in Care Settings,” Frontiers in Dementia 1 (2023): 1098446. 39081480 10.3389/frdem.2022.1098446 PMC11285665

[39] C. Corpus‐Espinosa , I. Mac Fadden , M. del Carmen Torrejón‐Guirado , and M. Lima‐Serrano , “Exploring Cultural Adaptations: A Scoping Review on Adolescent Mental Health and Substance Use Prevention Programs,” Prevention Science 26, no. 2 (2025): 204–221. 39888521 10.1007/s11121-025-01779-x PMC11891097

[40] J. Stoffregen and J. Pawlowski , “Culture Contextualization in Open E‐Learning Systems: Improving the Re‐Use of Open Knowledge Resources by Adaptive Contextualization Processes.” International Conference on Model‐Driven Engineering and Software Development (MODELSWARD) (IEEE, 2016), 767–774.

[41] K. L. Venner , A. Hernandez‐Vallant , K. A. Hirchak , and J. L. Herron , “A Scoping Review of Cultural Adaptations of Substance Use Disorder Treatments Across Latinx Communities: Guidance for Future Research and Practice,” Journal of Substance Abuse Treatment 137 (2022): 108716, 10.1016/j.jsat.2021.108716. 35148923 PMC9086178

[42] G. Bernal , “Intervention Development and Cultural Adaptation Research With Diverse Families,” Family Process 45, no. 2 (2006): 143–151. 16768015 10.1111/j.1545-5300.2006.00087.x PMC1693965

[43] NHS . Our Strategy ‐ Health Research Authority. n.d. https://www.hra.nhs.uk/about‐us/what‐we‐do/our‐strategy/.

[44] NIHR . Best Research for Best Health: The Next Chapter. 2021: 1–43. https://www.nihr.ac.uk/reports/best-research-for-best-health-the-next-chapter/34535.

[45] G. Bernal and C. Adames , “Cultural Adaptations: Conceptual, Ethical, Contextual, and Methodological Issues for Working With Ethnocultural and Majority‐World Populations,” Prevention Science 18, no. 6 (2017): 681–688. 28573426 10.1007/s11121-017-0806-0

[46] M. H. Shehadeh , E. Heim , N. Chowdhary , A. Maercker , and E. Albanese , “Cultural Adaptation of Minimally Guided Interventions for Common Mental Disorders: A Systematic Review and Meta‐Analysis,” JMIR Mental Health 3, no. 3 (2016): e5776. 10.2196/mental.5776 PMC5057065 27670598

[47] E. Heim and B. A. Kohrt , “Cultural Adaptation of Scalable Psychological Interventions,” Clinical Psychology in Europe 1, no. 4 (2019): 1–22.

[48] A. Secchi , A. Booth , I. Maidment , D. Sud , and H. Zaman , “Medication Management in M Inority, A Sian and B Lack Ethnic Older People in the United Kingdom: A Mixed‐Studies Systematic Review,” Journal of Clinical Pharmacy and Therapeutics 47, no. 9 (2022): 1322–1336. 35844186 10.1111/jcpt.13735

[49] F. Alhomoud , S. Dhillon , Z. Aslanpour , and F. Smith , “Medicine Use and Medicine‐Related Problems Experienced by Ethnic Minority Patients in the United Kingdom: A Review,” International Journal of Pharmacy Practice 21, no. 5 (2013): 277–287. 23418849 10.1111/ijpp.12007

[50] H. F. Sit , R. Ling , A. I. F. Lam , W. Chen , C. A. Latkin , and B. J. Hall , “The Cultural Adaptation of Step‐by‐Step: An Intervention to Address Depression Among Chinese Young Adults,” Frontiers in Psychiatry 11 (2020): 650. 32733296 10.3389/fpsyt.2020.00650 PMC7359726

[51] T. L. Cross , B. Bazron , K. W. Dennis , and M. R. Isaacs Towards a Culturally Competent System of Care. Vol. 1. 1989 Georgetown University Child Development Center, Washington, DC.

[52] C. J. Falicov , “Commentary: on the Wisdom and Challenges of Culturally Attuned Treatments for Latinos,” Family Process 48, no. 2 (2009): 292–309. 19579910 10.1111/j.1545-5300.2009.01282.x

[53] E. V. Cardemil , “Cultural Adaptations to Empirically Supported Treatments: A Research Agenda,” Scientific Review of Mental Health Practice 7, no. 2 (2010): 8–21.

[54] N. B. Wood , E. A. Erichsen , and C. L. Anicha , “Cultural Emergence: Theorizing Culture in and From the Margins of Science Education,” Journal of Research in Science Teaching 50, no. 1 (2013): 122–136.

[55] L. J. Parker , K. A. Marx , M. Nkimbeng , et al., “It's More Than Language: Cultural Adaptation of a Proven Dementia Care Intervention for Hispanic/Latino Caregivers,” Gerontologist 63, no. 3 (2023): 558–567. 35951488 10.1093/geront/gnac120 PMC10028233

[56] E. Heim and C. Knaevelsrud , “Standardised Research Methods and Documentation in Cultural Adaptation: The Need, the Potential and Future Steps,” Clinical Psychology in Europe 3, no. Spec Issue (2021): e5513. 36405674 10.32872/cpe.5513 PMC9670833

